# Physical and Rehabilitation Medicine Modalities in Pediatric Immune-Mediated Rheumatic Diseases: A Narrative Review of Current Evidence and Future Directions

**DOI:** 10.3390/jcm15218751

**Published:** 2026-11-09

**Authors:** Leonard Lamot, Frane Grubišić, Lovro Lamot

**Affiliations:** 1 Department of Physical and Rehabilitation Medicine, Zabok General Hospital and the Croatian Veterans Hospital, 49210 Zabok, Croatia; 2 Department of Rheumatology, Physical and Rehabilitation Medicine, Sestre Milosrdnice University Hospital Center, 10000 Zagreb, Croatia; 3 Department of Physical Medicine and General Rehabilitation, University of Zagreb School of Medicine, 10000 Zagreb, Croatia; 4 Department of Pediatrics, University Hospital Centre Zagreb, 10000 Zagreb, Croatia; 5 Department of Pediatrics, University of Zagreb School of Medicine, 10000 Zagreb, Croatia

**Keywords:** pediatric rheumatology, physical and rehabilitation medicine modalities, therapeutic exercise, hydrotherapy, electrotherapy, photobiomodulation, high-intensity laser therapy, TECAR therapy, juvenile idiopathic arthritis

## Abstract

Even when inflammation is controlled, impairments in strength, mobility, physical capacity, and participation may persist in children with immune-mediated rheumatic diseases. This narrative review examines therapeutic interventions relevant to physical and rehabilitation medicine (PRM) in multidisciplinary care. Literature identified through a narrative PubMed/MEDLINE search and examination of reference lists was synthesized by modality. Evidence was most extensive for land-based therapeutic exercise, particularly in juvenile idiopathic arthritis, whereas studies in juvenile dermatomyositis, childhood-onset systemic lupus erythematosus, and childhood-onset Takayasu arteritis were fewer and smaller. Aquatic therapeutic exercise has improved selected measures of pain, fitness, strength, fatigue, or function but has not demonstrated consistent superiority over well-designed land-based therapy. Digital delivery methods have shown potential to support access, feedback, and engagement within goal-directed, appropriately dosed programs. A small number of trials suggest possible adjunctive benefits of photobiomodulation, high-intensity laser therapy, and electromyographic biofeedback, whereas TECAR/CRET should remain investigational because direct evidence in pediatric rheumatic diseases is lacking. Individualized land-based therapeutic exercise should remain the core PRM modality, while aquatic therapeutic exercise may serve as an alternative or transitional option. Future trials should clarify prescription, dose, therapist involvement, adherence, adverse events, growth-plate safety, and participation outcomes.

## 1. Introduction

Immune-mediated rheumatic diseases in childhood affect a developing musculoskeletal system and may impair joint motion, muscle strength, cardiorespiratory fitness, gait, dexterity, and age-appropriate participation. Modern immunomodulatory treatment has greatly improved inflammatory control, yet fatigue, pain, deconditioning, fear of movement, glucocorticoid-related changes, and residual biomechanical deficits may persist. These problems remain clinically relevant in remission because childhood is the period in which movement competence, bone loading, fitness, and participation habits are established [1,2,3,4]. Current guidance for juvenile idiopathic arthritis (JIA) therefore includes physical and occupational therapy among recommended non-pharmacological interventions, although the certainty of evidence for individual procedures is generally low [5].

The terms physical activity, rehabilitation, and physical therapy are often used interchangeably, creating an imprecise evidence base. Physical activity includes all energy-consuming movement, whereas therapeutic exercise is a goal-directed rehabilitation intervention intended to improve fitness or function [1,6]. Rehabilitation is broader still and may include occupational therapy, orthoses, assistive devices, education, psychosocial interventions, and environmental adaptation. Here, physical and rehabilitation medicine (PRM) denotes a clinical field, not an eligibility criterion requiring documentation of a particular prescribing pathway. We include goal-directed therapeutic interventions even where research reports do not specify who prescribed them. In a typical multidisciplinary model, the pediatric rheumatologist defines disease activity, organ involvement, and medical restrictions, while the PRM specialist performs the functional assessment and prescribes the modality, therapeutic goals, treatment parameters, and precautions. The physiotherapist implements the prescription, monitors tolerance and response, and communicates the need for medical reassessment. Accordingly, orthoses, general sports participation, unsupervised physical training, and public-health activity promotion are not treated as independent PRM modalities, although progression toward safe autonomous activity remains an important therapeutic goal. Importantly, efficacy of an exercise program cannot by itself establish the effectiveness of a PRM-specialist-led service pathway.

Physical and rehabilitation medicine classifies treatment according to the physical or functional agent used. The principal PRM modalities considered here include land-based therapeutic exercise, hydrotherapy and aquatic therapeutic exercise, electrotherapy, photobiomodulation and high-intensity laser therapy, thermotherapy and cryotherapy, therapeutic ultrasound, manual techniques, and selected emerging modalities [6]. This review is organized by PRM modality to reflect clinical practice. The depth of coverage devoted to each modality is determined by the strength and breadth of the available pediatric evidence. The evidence is concentrated largely on active therapeutic exercise [7,8,9,10,11]. Electrotherapy, laser-based modalities, thermal agents, therapeutic ultrasound, manual techniques, and other externally applied procedures have been evaluated far less extensively [5,7,8,9,10,11]. The aim of this review is to critically synthesize the most relevant pediatric evidence by PRM modality, provide an indication-based framework for safe selection and progression, and identify areas where routine practice is based mainly on extrapolation rather than direct evidence.

## 2. Materials and Methods

### 2.1. Design and Scope

This structured narrative review was prepared with reference to the six SANRA domains [12]. It focused on children and adolescents with juvenile idiopathic arthritis (JIA), juvenile dermatomyositis (JDM), childhood-onset systemic lupus erythematosus (cSLE), juvenile systemic sclerosis, juvenile localized scleroderma, childhood vasculitides, and chronic nonbacterial osteomyelitis (CNO), including chronic recurrent multifocal osteomyelitis (CRMO). Eligibility was based on the intervention content, therapeutic goal, pediatric disease, and measurable outcomes, not on whether a particular specialist or physiotherapist was named in the published care pathway. Both supervised interventions and structured home programs were considered when their therapeutic aim was explicit. General promotion of physical activity, recreational sports, non-therapeutic wellness programs, and primary pain syndromes were excluded from direct disease-specific efficacy conclusions. Studies conducted exclusively in adults were not used to support direct pediatric efficacy conclusions. Observational studies were considered when they informed impairment, feasibility, safety, or evidence gaps.

### 2.2. Literature Search and Evidence Synthesis

PubMed/MEDLINE was the principal bibliographic source for the original narrative review (database inception to 6 October 2026); reference lists of relevant papers and guidelines were also examined. The original search documentation did not retain a verbatim executed search string, dated supplementary-query log, or record-by-record selection counts. To clarify the concepts used, a non-verbatim, reproducible conceptual search template is: (“Arthritis, Juvenile”[MeSH Terms] OR “juvenile idiopathic arthritis”[Title/Abstract] OR “juvenile dermatomyositis”[Title/Abstract] OR “childhood-onset lupus”[Title/Abstract] OR “juvenile systemic sclerosis”[Title/Abstract] OR “localized scleroderma”[Title/Abstract] OR “Takayasu arteritis”[Title/Abstract] OR “chronic nonbacterial osteomyelitis”[Title/Abstract]) AND (“Exercise Therapy”[MeSH Terms] OR “Physical Therapy Modalities”[MeSH Terms] OR “Hydrotherapy”[MeSH Terms] OR “therapeutic exercise”[Title/Abstract] OR “aquatic”[Title/Abstract] OR “laser”[Title/Abstract] OR “photobiomodulation”[Title/Abstract] OR “electrotherapy”[Title/Abstract] OR “biofeedback”[Title/Abstract] OR “telerehabilitation”[Title/Abstract] OR “TECAR”[Title/Abstract]). Emerging modalities were considered through targeted terms, but their exact original query dates were not retained. This reconstructed expression is provided to convey search concepts and must not be misinterpreted as the original executed query; exact supplementary dates and screening counts cannot be reconstructed reliably. The review did not systematically search Embase, PEDro, CINAHL, or other databases.

This is a structured narrative synthesis, not a systematic review. Original pediatric comparative studies were prioritized over uncontrolled reports for claims of effectiveness, and reviews or consensus documents informed context rather than counting as independent primary trials. The 19 principal studies summarized in Table 1 were selected to illustrate the major disease–modality combinations and important comparisons; this is a descriptive study table, not a complete screened-study denominator. Complete identification, deduplication, screening, and exclusion counts were not preserved and are therefore not reported as a retrospective PRISMA diagram. The appraisal considers randomization and comparator, sample size, blinding, intervention dose, follow-up, clinically relevant between-group effects, co-interventions, missing data, and whether adverse events were actively assessed. No formal risk-of-bias instrument or GRADE certainty assessment was performed; terms such as “multiple” and “limited” describe the amount of direct evidence, not validated certainty grades.

During manuscript preparation and revision, ChatGPT (OpenAI; GPT-5 and GPT-6) was used to support language editing, structural organization, preliminary presentation of study information, and the creation of schematic evidence and safety figures. Responsibility for verifying citations, study characteristics, figure classifications, and interpretation of the evidence rests with the authors.

## 3. Land-Based Therapeutic Exercise

Land-based therapeutic exercise has the broadest direct pediatric evidence base, particularly in JIA among PRM modalities in pediatric rheumatic diseases. Movement is used as the therapeutic agent through mobility work, progressive strengthening, aerobic conditioning, balance and proprioceptive training, neuromotor and functional practice, gait training, and targeted respiratory training. Most trials combine several of these elements. The evidence therefore supports the use of structured, goal-directed programs more strongly than any single training technique.

### 3.1. Mobility, Flexibility, and Functional Restoration

Range-of-motion activities, stretching, postural training, gait practice, and functional task practice are used to preserve movement and translate improvements in body function into daily activities. Their isolated effects are difficult to estimate because they are commonly embedded in programs with several components. In JIA, five systematic reviews indicate that structured therapeutic exercise and related physical therapy interventions can improve function and selected patient-reported outcomes without a consistent increase in disease activity, although protocols, comparators, and outcomes remain heterogeneous [7,8,9,10,11]. A randomized home program combining strengthening, stretching, posture, and functional activities improved physical function compared with usual care [13].

The same therapeutic principles are clinically relevant where direct trials are unavailable or insufficient to support conclusions for a specific disease. In juvenile systemic sclerosis, therapy commonly addresses reduced mobility of joints and soft tissues, contracture, hand function, posture, and, when required, orofacial or respiratory limitation [14]. Similar principles may be applied in juvenile localized scleroderma when skin or deeper tissue involvement restricts movement, although direct intervention evidence is insufficient. In CNO/CRMO, the practical goals are maintenance of mobility, graded loading, recovery of strength, and return to ordinary activity after pain and any lesion with high fracture risk have been medically assessed and controlled [15]. These applications are supported mainly by expert practice and extrapolation and are not assigned an efficacy estimate in this review.

### 3.2. Muscle Strengthening and Muscular Endurance

Progressive resistance work is intended to reverse weakness caused by inflammation, unloading related to pain, deconditioning, and glucocorticoid exposure. In JIA, vigorous aerobic and strengthening training was feasible without a clear increase in disease activity, although improvements were not uniform across outcomes [16]. Programs focused on strengthening, balance, and proprioception improved measures of lower extremity function, with differences between individual outcomes rather than a single superior approach [17]. A randomized trial of core stability training reported improvements in functional capacity and selected bone measures [18]. Small studies of slow-speed resistance training and longer programs designed to increase bone and muscle strength further support feasibility, but do not establish a universal dose or technique [19,20].

In clinically stable JDM, combined aerobic and resistance training improved selected muscle and functional outcomes without a clear signal of disease flare [21]. A twin case study and a home program similarly suggest that children with persistent weakness can adapt to training [22,23]. The evidence remains modest, and active myositis, dysphagia, calcinosis, contractures, compensatory movement, and bone vulnerability associated with glucocorticoid exposure require a lower initial dose and closer monitoring.

### 3.3. Aerobic and Cardiorespiratory Conditioning

Aerobic training is used to address deconditioning, reduced physical capacity, fatigue, and difficulty participating in daily, school, and recreational activities. The largest evidence base is again in JIA, where controlled trials and pooled analyses suggest gains in walking performance and selected fitness or functional outcomes, although effects on peak oxygen uptake and quality of life are inconsistent [7,8,9,10,11,16]. Aerobic work is also a component of successful JDM programs [21]. In inactive cSLE, supervised moderate aerobic training improved cardiorespiratory capacity and autonomic measures without worsening disease activity [24]. A small multicenter trial of home-based therapeutic exercise in clinically stable childhood-onset Takayasu arteritis suggested short-term feasibility in a mixed adolescent/young-adult sample (12–25 years) with stable disease; it does not establish safety in active vasculitis [25]. These findings should not be extrapolated to unstable cardiac or pulmonary disease, uncontrolled hypertension, major vascular ischemia, or active systemic inflammation.

### 3.4. Balance, Proprioception, and Training for Functional Tasks

Balance, proprioception, and practice of functional tasks are used when control of the lower limb, gait, dexterity, confidence, or participation is impaired. In JIA, Pilates improved selected measures of function and quality of life in one trial [26]. A later trial of clinical Pilates reported benefits in pain, cardiorespiratory fitness, function, and quality of life [27]. Training focused on balance and proprioception produced greater gains than strengthening for several measures of lower extremity function, but not for every outcome [17]. A small cSLE study of dual-task training that combined simultaneous motor and cognitive tasks suggested possible cognitive and quality of life benefits and requires replication [28]. These programs should be selected to address a defined functional deficit rather than offered as protocols branded for a specific disease.

### 3.5. Targeted Therapeutic Exercise and Digital Delivery

Digital platforms are methods for delivering therapeutic exercise rather than separate physical agents. Task-oriented training with Xbox Kinect (Microsoft Corporation, Redmond, WA, USA) improved activity performance and participation in one randomized JIA trial [29]. An internet program and a later study of gamified telerehabilitation demonstrated feasibility, but remote delivery did not ensure adherence or superior fitness outcomes [30,31]. Inspiratory muscle training is a targeted form of therapeutic exercise. A randomized JIA study reported improvements in selected cardiorespiratory measures [32]. A randomized trial published in 2026 compared two personalized programs delivered by physiotherapists. One used a home-based program, and the other used immersive virtual reality. Both groups improved, with differences between selected outcomes rather than consistent superiority of either delivery method [33]. An exploratory case series of action observation delivered through telerehabilitation also reported promising changes in motor and patient-reported outcomes, but the uncontrolled design precludes conclusions about efficacy [34]. Digital technologies, including telerehabilitation platforms, virtual reality, exergaming, and video-based action observation, may improve access, feedback, repetition, or engagement. They are delivery methods and may be integrated into clinically supervised, goal-directed programs and reassessed according to therapeutic goals, dose, and response.

### 3.6. PRM Prescription and Treatment Progression

No single frequency, intensity, time, type, volume, and progression prescription is supported across diseases. Across the included studies, prescriptions ranged from weekly aquatic programs to three or more supervised sessions per week and from approximately four weeks to six months [16,20,21,24,25,35,36,37]. This heterogeneity precludes a universal dose. After the pediatric rheumatologist has defined disease activity, organ involvement, and medical restrictions, the PRM specialist performs a functional assessment and prescribes the modality, therapeutic goals, initial dose, and relevant precautions. The physiotherapist implements the prescription, evaluates pain behavior, joint range, muscle performance, aerobic tolerance, and functional and participation goals, monitors the response, and progresses treatment within the prescribed plan. Pain during or after treatment, swelling, prolonged morning stiffness, disproportionate fatigue, loss of function, or systemic symptoms should prompt communication with the medical team and PRM reassessment rather than automatic abandonment of therapy [5,14,15,38].

## 4. Hydrotherapy and Aquatic Therapeutic Exercise

Hydrotherapy is a PRM modality and an aquatic environment in which prescribed therapeutic exercise can be delivered. Buoyancy reduces effective weight bearing, while water resistance permits graded movement and strengthening. Warm water may reduce perceived stiffness and facilitate movement [39]. These properties provide a clinical rationale for aquatic therapy but do not by themselves demonstrate consistent superiority over well-designed land-based therapeutic exercise [40,41].

### 4.1. Therapeutic Rationale and Principal Techniques

Aquatic programs may include mobility activities, walking or running in water, aerobic conditioning, resistance training, and, in selected children, plyometric training [35,36,37,42,43,44]. The intervention should be selected according to the therapeutic goal. Buoyancy can facilitate movement when loading on land is painful or poorly tolerated, whereas water resistance can increase muscular demand [39]. The pool should therefore be regarded as a controllable therapeutic environment in which different interventions can be delivered rather than as a uniform intervention.

### 4.2. Clinical Evidence and Outcomes

Most evidence concerns JIA. A multicenter randomized economic evaluation compared combined hydrotherapy and physiotherapy on land with techniques performed only on land. Adding hydrotherapy did not demonstrate a statistically convincing overall benefit compared with land treatment alone; the trial was under-recruited (78 rather than 200 planned participants) [40]. Smaller studies of aquatic fitness, water running, aquatic plyometric training, and underwater resistance combined with interferential current reported improvements in selected measures of pain, aerobic or anaerobic capacity, strength, bone properties, or physical fitness [35,36,42,43]. A trial of predominantly passive Watsu aquatic bodywork (not active aquatic exercise) reported benefits over the short term in selected outcomes, but evidence from a single study with a single-blind design remains insufficient to establish a distinct class effect [45]. A nonrandomized study published in 2026 reported improvements in both groups after intra-articular injection. It did not demonstrate superiority in global pain or functional outcomes, while exploratory findings in selected parent-reported quality-of-life domains suggested a possible association with added hydro-therapeutic exercise. Nonrandomized allocation, the common injection co-intervention, the absence of a valid between-group analysis, multiple unadjusted domain comparisons, and short follow-up preclude causal conclusions [46]. A systematic review concluded that hydrotherapy may reduce pain and improve maximal oxygen uptake compared with standard care while emphasizing the small number and heterogeneity of available studies [41].

Two small pediatric studies evaluated aquatic therapeutic exercise in JDM. A small crossover comparison of aquatic versus land exercise and a pilot study of aquatic plyometric training reported improvements in selected measures of function, fatigue, strength, or fitness [37,44]. These results support feasibility in carefully selected children but do not justify generalizing aquatic plyometric training to active myositis, marked weakness, substantial bone fragility, or unstable systemic disease.

### 4.3. Place in Clinical Practice

Aquatic therapy may be used as an alternative to land-based therapeutic exercise or as a transitional therapeutic environment when loading on land is poorly tolerated, but it is not an obligatory component of rehabilitation [40,41]. Its use should depend on the therapeutic goal, patient preference, and feasibility. Practical considerations include access to an appropriate pool, cost, transport, and the availability of trained supervision [40]. Suitability for pool treatment should be assessed individually, with attention to active infection, impaired skin integrity, draining wounds, continence, and conditions that affect temperature regulation [39]. Recreational swimming and balneotherapy or spa treatment are distinct from prescribed aquatic therapeutic exercise, and evidence relating to one intervention should not be extrapolated to another.

## 5. Electrotherapy

Electrotherapy encompasses currents with different physiological targets, and pediatric rheumatology evidence is too sparse to support a broad class effect. In one randomized JIA study, interferential current was combined with resistive underwater training. Favorable outcomes cannot be attributed confidently to the current because the active training component also differed [43]. In another trial, transcutaneous electrical nerve stimulation (TENS) was included in a multimodal comparator with hot packs and strengthening, so its isolated contribution remains unknown [47]. A randomized study of electromyographic (EMG) biofeedback during quadriceps training reported greater improvements than conventional therapy alone [48]. This finding supports biofeedback as an adjunct that provides feedback during active movement. It should not be interpreted as evidence for a general electrotherapy class effect. Direct pediatric evidence for neuromuscular or functional electrical stimulation in immune-mediated rheumatic disease remains insufficient.

Accordingly, if electrotherapy is used selectively for short-term symptom modulation, it should remain adjunctive and should not displace progressively dosed movement. Future trials should compare an identical therapeutic exercise program with and without the electrical modality, report electrode placement and current parameters completely, and include outcomes beyond immediate pain relief.

## 6. Phototherapy and Laser Therapy

Laser-based modalities warrant more attention than most externally applied PRM modalities because two pediatric randomized studies directly evaluated them as adjuncts to therapeutic exercise. In 60 children with polyarticular JIA, photobiomodulation/low-level laser therapy (PBM/LLLT) applied to the knee (EL12061 FISIOLASER IRD; Chinesport S.p.A., Udine, Italy; 903 nm, 50 mW, 1.5 J) plus therapeutic exercise produced more favorable trajectories for pain, fatigue, and functional status than therapeutic exercise alone, but did not improve quadriceps peak torque [49]. A second small study evaluated pulsed high-intensity laser therapy (HILT) using a neodymium-doped yttrium aluminum garnet (Nd:YAG) laser (HIRO 3; ASA, Arcugnano, Vicenza, Italy) in children classified as having juvenile rheumatoid arthritis in the original report and found greater improvements in pain and gait-related outcomes than placebo laser when both groups also received therapeutic exercise [50].

PBM/LLLT and HILT differ substantially in power, energy delivery, tissue effects, and dosimetry and should not be treated as a single intervention category [49,50]. These two small studies suggest possible short-term analgesic or functional effects when laser is used as an adjunct but do not support replacement of active therapeutic exercise. Both studies were small, examined selected joints and outcomes, and were not designed to assess uncommon or long-term safety outcomes. Future studies should report wavelength, power, energy or energy density, pulse characteristics, treated area, number of sessions, co-interventions, and adverse events.

Particular caution is required in skeletally immature children. Experimental evidence concerning direct exposure of open physes is inconsistent: one rat study found no changes in growth-plate structure or final limb length after PBM/LLLT at the tested parameters [51], whereas another reported adverse histomorphometric effects and reduced longitudinal growth after irradiation of the distal femoral physis [52]. These findings cannot be directly extrapolated to treatment in children, and the magnitude of any treatment-related risk remains unknown. A precautionary approach may include avoiding unnecessary direct irradiation over open growth plates, documenting dosimetry carefully, and assessing growth-related and long-term safety outcomes. In growing children, the laser modality and treatment parameters should be prescribed by a PRM specialist after an individualized benefit-risk assessment.

## 7. Thermal, Manual, and Emerging PRM Modalities

Superficial heat, paraffin, local cryotherapy, massage, manual therapy, and therapeutic ultrasound are familiar PRM modalities [6]. Transfer of energy capacitive and resistive (TECAR), also termed capacitive-resistive electric transfer (CRET), is an emerging radiofrequency diathermy modality. One pediatric randomized trial evaluated TECAR combined with biofeedback for fecal incontinence [53], but no direct pediatric studies were identified in the immune-mediated rheumatic diseases covered by this review. This absence of direct disease-specific evidence is not evidence of inefficacy. Extrapolating efficacy from the fecal-incontinence trial would be inappropriate, particularly because disease-specific dosing, thermal monitoring, and long-term safety in developing tissues remain undefined. A single case report described improvement after orthopedic manual physical therapy combined with therapeutic exercise, but it cannot separate treatment effects from natural variation or the additional intervention [54]. A small older massage trial suggested symptomatic benefit over the short term but is insufficient to define efficacy or dose [55]. The literature search did not identify controlled pediatric studies with sufficiently detailed intervention protocols and outcome reporting to support conclusions about paraffin therapy, therapeutic ultrasound, extracorporeal shock wave therapy, dry needling, or whole-body vibration in the target diseases. The available evidence therefore does not permit reliable conclusions about efficacy, safety, or treatment dose.

**Figure 1:**
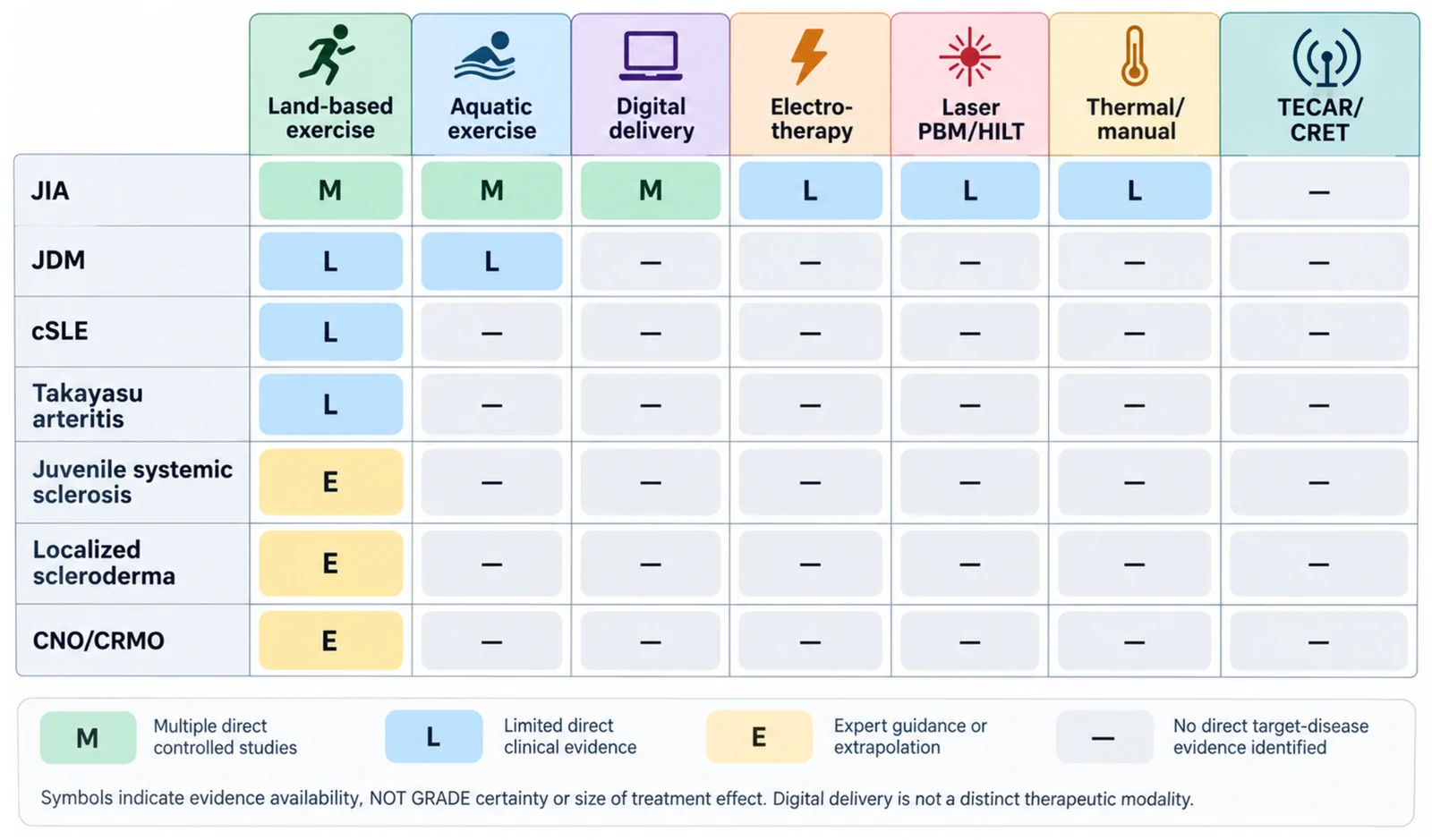
Disease-by-modality map of direct pediatric rehabilitation evidence. M denotes multiple direct controlled studies, L limited direct evidence, E expert guidance/extrapolation, and a dash indicates no direct evidence identified in the sources reviewed. Categories do not represent formal GRADE certainty.

Established but poorly studied modalities such as superficial thermal agents or manual techniques, if used, should have only a limited adjunctive role in short-term symptom management and should be selected according to a defined treatment goal and patient preference [6,54,55]. Given the limited pediatric evidence, they should not be presented as having an evidence base comparable to land-based or aquatic therapeutic exercise. TECAR/CRET should be regarded as a research priority rather than a routine clinical option in pediatric rheumatology. Claims for all of these modalities should remain proportionate to the available evidence and should not imply durable improvements in function or participation. The disease-specific distribution of evidence and limitations is summarized in Table 2 and Figure 1.

**Table 1:** Selected principal intervention reports (*n* = 19) and study-level limitations. The table is an illustrative evidence summary, not a complete screening denominator; numerical comparisons and safety ascertainment are stated only where identified in available source information. NR = not reported or not independently confirmed in the consulted record.

Study; Disease/Sample	Intervention and Comparator	Treatment/Follow-Up	Reported Outcome (Type of Contrast)	Design and Interpretation/Safety
[13] Tarakci et al.; JIA, *n* = 81	Individualized exercise, 4 home days/week + 1 hospital session/week vs. waiting list (*n* = 43 vs. 38)	12 weeks	Most functional/QoL changes favored exercise; pain change did not differ significantly between groups	Single-blind RCT; age NR in abstract; short follow-up
[16] Singh-Grewal et al.; JIA, *n* = 80, 8–16 y	High-intensity aerobic training vs. qigong; 3×/week	12 weeks	No between-group difference in VO2submax or other exercise-test measures; both groups improved C-HAQ	Well tolerated; active comparator; adherence lower with high-intensity training
[21] Habers et al.; JDM, *n* = 26, 8–18 y	Individualized treadmill interval + strength vs. waiting-list usual care	12 weeks + 12-week follow-up	Standing long jump +13 cm (95% CI 2–23), push-ups +8 (3–13), sit-ups +4 (0.4–8), parent CHAQ −0.13 (−0.24 to −0.01) vs. control	75% completed; reported reasons for discontinuation included fatigue/motivation, recurrent infections and complaints; 12-week follow-up
[24] Prado et al.; cSLE, *n* = 19	Supervised moderate aerobic training vs. non-trained control	12 weeks	Time-to-exhaustion, peak speed, peak VO2 and HR recovery improved vs. control; SLEDAI-2K stable	Small RCT; short-term stable/inactive population
[25] Astley et al.; childhood-onset Takayasu, *n* = 14, 12–25 y	Home exercise vs. standard care in remission	12 weeks	No worsening of arterial inflammation; selected physical activity/function/body composition outcomes favored exercise	Very small mixed adolescent/young-adult sample; remission only
[28] Kisa et al.; cSLE, *n* = 30	Dual-task motor/cognitive exercise, 2×/week; uncontrolled pre/post study	16 weeks	MoCA 20.86 ± 3.89 to 23.73 ± 2.72; McGill pain 42.70 ± 16.85 to 36.53 ± 16.55 (within-group only)	No comparison group; improvement is associative, not evidence of causal efficacy; safety monitoring NR
[29] Arman et al.; JIA, *n* = 62	Xbox Kinect task-oriented training vs. real-material task practice; 3×/week	8 weeks	Both improved; video-game group superior for several upper-limb strength, satisfaction and hand-function outcomes	Delivery-method comparison; not evidence for VR as a unique physical agent
[30] Armbrust et al.; JIA, *n* = 49	Internet program + 4 group sessions vs. control	Intervention with 3- and 12-month follow-up	Within-group activity/exercise-capacity gains, but no significant between-group differences	Important example of feasibility/change without comparative superiority
[32] Sarac et al.; JIA, *n* = 33, 13–18 y	Home inspiratory muscle training (*n* = 17) vs. no added treatment (*n* = 16); 2 × 30 breaths/day; start at 60% PImax	8 weeks; dose increased 10% of starting load every 2 weeks	Between-group change favored IMT for FVC, FEV1, PImax, VO2peak and METs (*p* < 0.05)	Small RCT; achieved adherence ≈ 68%; no adverse effects reported in the trial, limited follow-up
[33] Arman et al. 2026; JIA, *n* = 50, 13–18 y	HomeEX vs. immersive JiaFitXR (2×/week, 25–30 min/session)	8 weeks	Both improved; VR favored selected lower-limb outcomes, home exercise favored flexibility/upper-limb strength	Single RCT; participants with active arthritis excluded; dropout HomeEX 9/25 and VR 7/25; LOCF imputation; follow-up limited
[35] Takken et al.; JIA, *n* = 54, 5–13 y	Supervised aquatic fitness 1 h/week vs. control	≈20 sessions	No statistically significant between-group differences in functional, QoL, joint or fitness outcomes	RCT; no worsening in health status reported; low treatment frequency
[37] Samhan et al.; JDM, *n* = 14, 10–16 y	Aquatic vs. land therapeutic exercise, each 45 min 2×/week, crossover (*n* = 14)	4 weeks per intervention period; crossover	Aquatic favored hip-flexor strength (*p* = 0.007), hip-abductor strength and fatigue/skin score; no clear between-modality advantage for shoulder strength	Very small crossover; only short-term outcomes; no estimate of rare adverse events
[40] Epps et al.; JIA, *n* = 78 recruited (planned *n* = 200), age 4–19 y	Combined aquatic + land PT vs. land PT alone; 16 one-hour sessions in 2 weeks, then local PT	2-week intensive intervention; 2- and 6-month outcomes	Disease response at 2 mo: 47% combined vs. 61% land; no demonstrated clinical or cost/QALY advantage of adding aquatic care	Under-recruited multicenter RCT, active comparator; cannot rule out moderate benefits; exacerbations examined
[44] Elnaggar and Abd El-Nabie; JDM, *n* = 16, mean age 13.44 y	Aqua-plyometric exercise vs. standard outpatient care, single-blind randomized crossover	Two treatment periods separated by 1-month washout; period duration NR in abstract	Aquatic favored selected hip/knee strength (*p* < 0.001 for several measures), fatigue (*p* < 0.001), function (*p* = 0.009) and activity (*p* = 0.0001)	Tiny crossover pilot; no long-term safety inference; intervention-specific dosing requires full-text confirmation
[45] Pérez Ramírez et al.; JIA, *n* = 46, 8–18 y	Predominantly passive Watsu aquatic bodywork vs. active conventional aquatic exercises; 45 min/week	10 sessions + 3-month follow-up	Selected HRQoL, disability and functional-health outcomes favored Watsu	Single-blind RCT; heterogeneous active/passive interventions; requires independent replication
[46] Gnasso et al.; JIA, *n* = 30, ages 3–17 y (22 oligoarticular)	Hydro-therapeutic exercise after intra-articular steroid injection vs. injection without added hydrotherapy	Before intervention and 1-month reassessment	No significant overall between-group advantage in global CHAQ or VAS; exploratory differences in selected parent-reported QoL domains	Non-randomized design, universal injection co-treatment, small study; exploratory domain comparisons and confounding
[48] Eid et al.; polyarticular JRA/JIA, *n* = 36, 8–13 y	EMG-biofeedback isometrics + conventional therapy vs. conventional therapy; 3×/week	12 weeks	At 12 weeks, pain, quadriceps strength and function improved more with biofeedback	Small RCT; historical disease label; adjunctive effect only
[49] Elnaggar et al.; polyarticular JIA, *n* = 60	PBM/LLLT 903 nm, 50 mW, 1.5 J + exercise vs. exercise alone	Treatment + 6-month follow-up	Pain *p* = 0.003, fatigue *p* = 0.004, function *p* = 0.022 favored PBM; no torque difference	Single trial; selected joint/outcomes; not powered for uncommon long-term harms
[50] El-Shamy et al.; JRA/JIA, *n* = 30, mean 10.5 y	Pulsed Nd:YAG HILT + exercise vs. placebo HILT + same exercise; 3×/week	4 weeks + 12-week follow-up	Pain and gait outcomes favored HILT at post-treatment and follow-up	Small single trial; historical label; long-term growth/safety not established

**Table 2:** Disease-specific rehabilitation evidence and clinical precautions. Direct evidence, guidance, and extrapolation are deliberately distinguished.

Disease	Direct Rehabilitation Evidence	Common Functional Targets	Disease-Specific Precautions	Interpretation
JIA	Largest evidence base: multiple controlled land-based and aquatic exercise studies; selected digital, electro/biofeedback, and laser trials [7,8,9,10,11,12,13,16,17,18,19,20,26,27,28,29,30,31,32,33,34,35,36,40,41,42,43,44,45,46,47,48,49,50]	Pain-limited function, mobility, strength, aerobic/anaerobic capacity, gait, balance, participation	Active synovitis, joint damage, pain behavior, glucocorticoid/bone effects; tailor load to involved joints	Most conclusions in this review are strongest for JIA, but no single modality or dose is universally superior
JDM	One small land-based RCT plus case/home studies; two small aquatic crossover/pilot studies [21,22,23,37,44]	Proximal strength, fatigue, aerobic capacity, function, participation	Active myositis, severe weakness, dysphagia, calcinosis, contracture, steroid-related bone vulnerability	Direct evidence exists but is small; lower starting dose and close monitoring are often appropriate
cSLE	One small aerobic RCT; one uncontrolled dual-task study [24,28]	Aerobic capacity, fatigue, cognition, daily participation	Disease activity, nephritis/renal status, hypertension, cardiopulmonary disease, thrombosis risk, glucocorticoid effects	Evidence supports selected exercise in stable patients; do not generalize to active organ-threatening disease
Childhood-onset Takayasu arteritis	One very small RCT in remission with participants aged 12–25 years [25]	Deconditioning, activity, function, cardiometabolic health	Vascular ischemia/aneurysm, hypertension, cardiac involvement, active arterial inflammation	Promising feasibility only; mixed-age sample and remission-only design limit pediatric generalization
Juvenile systemic sclerosis	No controlled PRM intervention trials identified; expert guidance [14]	Joint/soft-tissue mobility, contracture, hand function, posture, orofacial/respiratory function	Skin integrity, digital ischemia/Raynaud phenomenon, cardiopulmonary disease, GI/nutritional status	Recommendations are guidance/extrapolation rather than demonstrated rehabilitation efficacy
Juvenile localized scleroderma	No disease-specific controlled intervention evidence identified	Mobility when skin/deep tissue disease restricts motion, contracture prevention, function	Depth and activity of skin/fascial involvement; wound/skin tolerance	Functional treatment may be reasonable, but efficacy is unproven
CNO/CRMO	No controlled PRM efficacy trials identified; clinician guidance [15]	Mobility, graded loading, strength, return to school/sport	Pain source, spinal/long-bone lesions, fracture risk, active inflammatory lesions	Use lesion-specific medical assessment before loading; recommendations are mainly extrapolated

## 8. Safety, Indications, and Individualization

The central principles of safe rehabilitation are to identify a specific functional indication, evaluate disease activity and relevant organ damage, select a modality and starting dose appropriate to the child, define monitoring endpoints, and reassess promptly if symptoms or function deteriorate. Safety depends more on the child, disease state, organ involvement, and dose than on the diagnostic label alone. Guidance for specific diseases supports individualized rehabilitation, maintenance of mobility and strength, and avoidance of unnecessary deconditioning. It also requires adaptation to active inflammation and organ involvement [5,14,15,38]. Before treatment, the pediatric rheumatologist and PRM specialist should consider active synovitis, myositis, tendon or joint damage, contracture, bone density and fracture risk, cardiovascular or pulmonary involvement, hypertension, neuropathy, skin lesions, infection risk, medication effects, and any modality-specific precaution. Children with stable disease should generally be protected from deconditioning rather than routinely restricted. PRM treatment does not routinely need to be withheld until all inflammation has resolved. During a flare, the aim and dose are modified toward comfortable range of motion, isometric muscle activation, positioning, respiratory care, and maintenance of essential function, while high-impact activity and heavy loading of actively inflamed tissues are reduced. Progression resumes as inflammation and systemic status permit. Stable weakness, reduced aerobic capacity, stiffness, loss of mobility, or restricted participation may indicate graded therapeutic exercise. Active synovitis generally calls for reducing joint load and impact rather than withdrawing all movement. Severe myositis or systemic disease requires a lower initial dose and closer medical supervision. These are pragmatic clinical principles supported variously by pediatric studies, guidelines, and expert inference, not validated universal thresholds [5,14,15,38].

**Figure 2:**
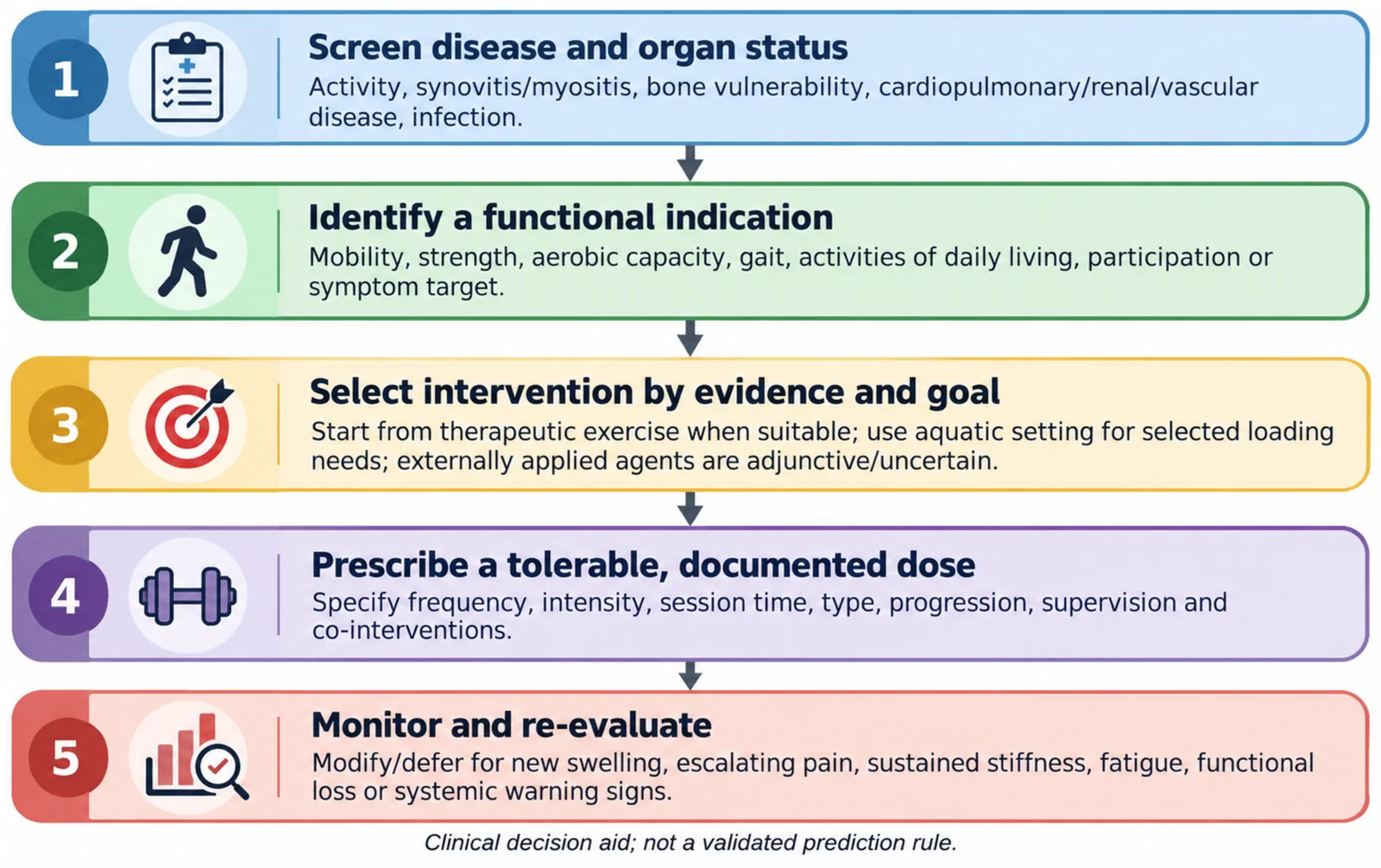
Indication-based safety and progression framework. This is a practical synthesis of pediatric studies, disease guidance, and clinical precautions rather than a validated clinical prediction rule.

Table 3 specifies indications, precautions, evidence sources, and reasons to modify or defer treatment. Figure 2 presents the clinical decision sequence. These clinical safeguards reflect a mixture of direct pediatric data and expert reasoning; they are not formal contraindication rules.

## 9. Discussion

The available evidence is unevenly distributed across PRM modalities and pediatric rheumatic diseases. Land-based therapeutic exercise has the broadest evidence base and the greatest clinical applicability. Aquatic therapy has been evaluated mainly in JIA and, to a lesser extent, JDM. Direct evidence for electrotherapy, PBM/LLLT, HILT, thermal agents, therapeutic ultrasound, and manual techniques is substantially more limited. Newer studies describe individualized active programs delivered in varied clinical settings, but do not establish a particular specialist-led service pathway, while broadening delivery options through virtual reality, telerehabilitation, action observation, and biofeedback. It does not overturn the central hierarchy of evidence. Table 4 summarizes the strength of evidence, clinical role, and research status of the principal modalities included in this review. Greater trial volume in JIA may reflect research concentration rather than an intrinsically greater effect of exercise in that disease. Many trials combine strengthening, aerobic training, education, and symptom management, preventing attribution to any one component; short-term improvement within the intervention group is not equivalent to superiority over an active comparator. Most outcome measures assess pain, strength, range, or fitness; consistent long-term evidence for school participation, attendance, social participation, and quality of life is much less developed. Our addition is a disease-by-modality evidence map and an indication-based safety framework rather than a pooled estimate of efficacy across heterogeneous diagnoses.

The resulting hierarchy is clinically straightforward. Active therapeutic exercise has the largest body of direct evidence, predominantly in JIA, and aquatic therapeutic exercise remains an active PRM modality delivered in a distinct physical environment. PBM/LLLT and HILT are the most directly studied externally applied modalities, but their evidence base remains narrow and long-term pediatric safety data are limited. EMG biofeedback has one supportive trial as an adjunct that provides feedback during strengthening. Evidence for generic electrotherapy, thermal agents, massage, and manual procedures remains limited. TECAR/CRET has no direct evidence in the target pediatric rheumatic diseases and should be treated as unproven in the target diseases, rather than assumed ineffective.

## 10. Limitations of the Review

This narrative review has several limitations. The search was limited to PubMed/MEDLINE and examination of reference lists, so relevant studies indexed elsewhere may have been missed. The evidence base was dominated by small, single-center studies focused on JIA, with heterogeneous interventions and incomplete reporting of prescription, dose, adherence, co-interventions, and adverse events. Variation in PRM and physical therapy terminology across countries and disciplines further complicated the classification of interventions. Therefore, the absence of adequate pediatric evidence for a modality should be interpreted as uncertainty rather than evidence of no effect. In addition, the original search history lacks a reproducible verbatim query and item-level screening log, and searches were not systematically extended to rehabilitation-specific databases. No prespecified eligibility register, formal risk-of-bias evaluation, or GRADE certainty assessment was undertaken. Purposive selection may have missed relevant negative studies, and the studies highlighted in Table 1 are illustrative rather than an exhaustive set. Evidence availability in Figure 1 must not be confused with certainty of benefit. Where adverse events were not explicitly evaluated, their absence from a report cannot establish safety.

## 11. Research Priorities

- Use multicenter designs and adequately powered pediatric samples, especially outside JIA.
- Report the complete frequency, intensity, time, type, volume, and progression of treatment, together with therapist contact, additional interventions, adherence, and contamination.
- Separate disease activity from impairment and include participation, school, sport, fatigue, and outcomes prioritized by patients.
- For externally applied modalities, compare identical therapeutic exercise programs with and without the modality so that its additional benefit can be estimated.
- Report flares and adverse events explicitly and include assessment after supervised therapy ends.
- Standardize PBM/LLLT and HILT dosimetry and electrotherapy parameters, and prospectively assess exposure near open physes and long-term safety.
- Evaluate emerging modalities such as TECAR/CRET only in prospectively designed pediatric studies with appropriate comparators, thermal monitoring, and long-term safety assessment before routine use.

## 12. Conclusions

Within multidisciplinary care, individualized land-based therapeutic exercise can be a central rehabilitation intervention when selected by clinical need and delivered within multidisciplinary care for children with immune-mediated rheumatic diseases. Aquatic therapeutic exercise is a credible alternative or transitional option when loading, pain, or weakness limits treatment on land, but it has not proved uniformly superior. PBM/LLLT and HILT have shown possible short-term adjunctive benefit in individual small trials in JIA, but the trials are few and long-term safety data in growing children are inadequate; treatment near open physes therefore requires particular caution. EMG biofeedback may support targeted strengthening. Direct pediatric evidence remains inadequate for generic electrotherapy, thermal agents, massage, manual therapy, therapeutic ultrasound, and other externally applied procedures. TECAR/CRET has no direct evidence in the target diseases and should remain a research priority rather than a routine clinical option. This conclusion is strongest for JIA, more tentative for JDM and cSLE, and largely based on extrapolation for juvenile systemic sclerosis, juvenile localized scleroderma, most childhood vasculitides, and CNO/CRMO.

## Figures and Tables

**Table 3:** Indications, modifications, and clinical reassessment criteria.

Clinical Situation	Indication/Treatment Goal	Modification or Precaution	Reassess/Defer When
Stable disease with deconditioning, weakness, or restricted mobility	Progressive therapeutic exercise to restore capacity and participation	Start from measured baseline and progress by tolerance; avoid unnecessary restriction [5]	New inflammatory or systemic features, persistent functional decline
Active synovitis or marked joint pain	Comfortable motion, isometric/submaximal activation, maintenance of essential function	Reduce impact and heavy loading of actively inflamed joints; coordinate inflammation control [5]	New swelling, prolonged morning stiffness, escalating post-treatment pain
Active myositis or severe weakness	Maintain safe movement, submaximal activation, respiratory/functional goals	Lower initial dose, longer recovery, avoid compensatory overload; monitor dysphagia/calcinosis and fatigue [38]	Disproportionate fatigue, functional loss, systemic worsening
Glucocorticoid exposure or low bone mass	Gradual restoration of strength and bone-loading capacity	Avoid abrupt high-impact progression; assess fracture risk and introduce resistance/loading gradually [5,38]	Focal bone pain, suspected fracture, tendon injury
Cardiac, pulmonary, renal, hypertensive, or vascular involvement	Exercise only when the organ-specific status permits a defined target	Obtain disease-appropriate medical clearance; monitor symptoms and objective response [14,25,38]	Chest pain, syncope, abnormal BP response, ischemic symptoms, worsening organ function
Skin disease, wounds, infection risk, or pool-related concerns	Aquatic or device-based treatment only when skin/infection status permits	Inspect skin; adapt or defer pool, taping, heat, or electrodes as appropriate [14,38,39]	Active infection, draining wound, impaired skin integrity, poor temperature regulation
PBM/LLLT or HILT in a skeletally immature child	Only for a defined adjunctive indication when expected benefit outweighs uncertainty	Avoid unnecessary direct irradiation over open physes; document full dosimetry and follow-up [49,50,51,52]	Unexpected pain, tissue reaction, or any concern about growth-related effects
Pain amplification or fear of movement	Graded exposure, education, achievable functional goals	Interpret pain in context of tissue status; coordinate messaging across the team [5]	New objective inflammatory signs or progressive loss of function

Note. The recommendations combine direct pediatric evidence, clinical guidance [5,14,15,38,39,49,50,51,52], and explicit precautionary reasoning. They are not validated universal contraindications. Clinical decisions must account for disease activity and organ involvement.

**Table 4:** Evidence availability and cautious clinical role of principal interventions.

Intervention/Approach	Direct Pediatric Evidence	Evidence Characterization	Clinical Interpretation
Land-based therapeutic exercise	JIA trials [13,16,17,18,19,20,26,27]; JDM [21,22,23], cSLE [24,28], childhood-onset Takayasu [25]; reviews [7,8,9,10,11]	Multiple direct controlled studies, but heterogeneous by disease, comparator, dose, and outcome	Best-supported core rehabilitation intervention. Do not infer one universal exercise protocol or a specific professional prescribing pathway from intervention trials.
Aquatic therapeutic exercise	JIA controlled studies [35,36,40,42,43,46]; small JDM crossover/pilot reports [37,44]; review [41]	Direct evidence exists, but comparative results are inconsistent and often small or heterogeneous	May be useful when land loading is poorly tolerated or for a specific goal. Consistent superiority over well-designed land exercise is not established.
Passive aquatic bodywork (Watsu)	One JIA randomized active-comparator trial (*n* = 46) [45]	Limited direct evidence; different therapeutic content from active aquatic exercise	Selected patient-reported improvements; one trial is insufficient for a distinct class recommendation.
Digital delivery: telerehabilitation, VR, exergaming, action observation	Controlled JIA studies plus a small exploratory case series [29,30,31,32,33,34]	Evidence ranges from comparative effectiveness to feasibility only	Treat as delivery approaches, not distinct physical agents. Separate access/engagement/feasibility from clinical superiority.
EMG biofeedback/other electrotherapy	One supportive EMG-biofeedback RCT; other electrical interventions confounded by multimodal programs [43,47,48]	Limited direct evidence	Biofeedback may be an adjunct to active exercise. Generic electrotherapy class effects are not established.
PBM/LLLT	One 60-participant JIA RCT [49]; preclinical physis studies inconsistent [51,52]	Limited direct efficacy evidence; long-term safety uncertainty	Possible short-term adjunctive benefit for pain, fatigue, and function. Avoid broad routine recommendations; growth-plate precautions are inferred from preclinical uncertainty.
HILT	One 30-participant randomized JIA/JRA trial [50]	Very limited direct evidence; long-term safety uncertain	Possible adjunctive benefit for pain/gait in a selected population. Independent replication and standardized dosimetry are needed.
Superficial thermal agents and manual techniques	Case report of manual treatment [54], small massage trial [55]; not an intervention class trial	Sparse clinical evidence; multimodal confounding and short follow-up	May be considered only for a defined short-term symptom goal; durable functional efficacy and specific dose remain uncertain.
TECAR/CRET (radiofrequency diathermy)	No direct pediatric rheumatology efficacy study among cited sources; one pediatric fecal-incontinence study [53] is outside the target diseases	No direct target-disease evidence (not an established negative efficacy result)	Investigational for these diseases. Establish comparative efficacy, disease-specific parameters, thermal monitoring, and long-term safety before routine use.

## Data Availability

No new data were created or analyzed in this study. Data sharing is not applicable to this article.

## Abbreviations

The following abbreviations are used in this manuscript:

CNO	chronic nonbacterial osteomyelitis
CRET	capacitive-resistive electric transfer
CRMO	chronic recurrent multifocal osteomyelitis
cSLE	childhood-onset systemic lupus erythematosus
EMG	electromyographic
HILT	high-intensity laser therapy
JDM	juvenile dermatomyositis
JIA	juvenile idiopathic arthritis
LLLT	low-level laser therapy
Nd:YAG	neodymium-doped yttrium aluminum garnet
PBM	photobiomodulation
PRM	physical and rehabilitation medicine
TECAR	transfer of energy capacitive and resistive
TENS	transcutaneous electrical nerve stimulation
